# X-ray data processing

**DOI:** 10.1042/BSR20170227

**Published:** 2017-10-06

**Authors:** Harold R. Powell

**Affiliations:** Harry Powell Crystallographic, Buxton Road, Chingford, London E4 7DP, U.K.

**Keywords:** crystallography, data processing, software

## Abstract

The method of molecular structure determination by X-ray crystallography is a little over a century old. The history is described briefly, along with developments in X-ray sources and detectors. The fundamental processes involved in measuring diffraction patterns on area detectors, i.e. autoindexing, refining crystal and detector parameters, integrating the reflections themselves and putting the resultant measurements on to a common scale are discussed, with particular reference to the most commonly used software in the field.

## Introduction

The vast majority of detailed 3D structural information that has been discovered about proteins has been from single crystal X-ray diffraction studies (‘X-ray crystallography’); a review in this journal noted recently that over 120000 molecular structures have been deposited into the Protein Data Bank since its establishment in 1971 [1].

This report is concerned with the methods developed for measuring the intensities of diffraction maxima (‘reflections’ or ‘spots’) in the recorded images over the last few decades, and with the current state of affairs. The quality of the images is paramount; while the developments in data processing have been significant, it is often forgotten that X-ray data collection is the last experiment in a process that may well have taken months or years to reach the point where suitable crystals are available, and some care should be taken in this final step. If the data collected are particularly bad, it is likely that no processing program will be able to integrate the dataset.

## Historical background

Since its discovery just over a century ago, the method of single crystal X-ray diffraction has become the gold standard in determining the structure of both small and macromolecules (e.g. proteins). The first inorganic compounds (a group of alkali metal halides) and diamond had their 3D structures determined in 1913 [2,3], the first organic compound (hexamethylene tetramine) in 1923 [4] and the first protein (myoglobin) in 1958 [5]. While early measurements of the diffraction maxima used scintillation counters (recording the intensities of data one by one) or X-ray sensitive film, since the 1980s electronic area detectors have been used because of their superior characteristics, e.g. recording directly on to the digital media, higher sensitivity and lower intrinsic background. Originally the electronic detectors were devices such as television-type cameras or multiwire proportional counters (late 1970s to early 1990s), but the major advance was the introduction of image plates and charge-coupled devices (late 1980s to mid 1990s). More recently, these have been superseded by pixel array detectors (‘PAD’s, mid 2000s to present) using either photon counting [6] or charge integrating [7] technologies.

While other methods exist (see at the end of this report), the Arndt–Wonacott rotation method of data collection [8], has underpinned the use of X-ray diffraction in biological structure determination. It allows systematic sampling of the diffraction from monocrystalline samples, and since 1990s, this has been the method of choice for single crystal studies. The technique involves rotating a crystal about an axis, ideally perpendicular, to an illuminating monochromatic X-ray beam, and the diffracted X-rays are detected on an area detector. In many cases, a single experiment efficiently samples the X-ray diffraction volume and the resulting datasets are often complete, with many equivalent data points sampled multiple times. For high resolution datasets or those where the crystal lacks internal symmetry, there may be systematically missing regions of data, and the method needs to be modified by collecting further data after reorienting the crystal with respect to the rotation axis or rotating the crystal about a different diffractometer axis.

At the same time when the detectors were being improved, X-ray sources were also undergoing major developments. Until the middle of the 1970s, monochromatic X-ray sources were essentially limited to either sealed tubes or rotating anode generators, both of which had been known since the mid-1910s. Synchrotron radiation from electrons circulating in particle storage rings was first identified in 1947 [9] as something of a nuisance by the physicists operating the devices, who were mainly interested in the particles themselves, but in 1972 a project was started to construct an X-ray beamline at the Stanford University storage ring, SPEAR, to provide more intense X-rays than could be produced in a home laboratory; this resulted in the first experiments into protein crystallography at the site a few years later [10]. Globally, the first dedicated synchrotron light source was the SRS at Daresbury Laboratory in the U.K., where the production of X-rays (in particular) was the primary purpose of the instrument. This became available to users in 1981, and allowed the study of smaller samples and the collection of better data than previously, on wavelength tuneable beamlines [1]. Developments across the globe since then have given more intense and better focused X-rays allowing the analysis of crystals a few microns across, as well as the use of specific wavelengths to utilize effects arising from atomic X-ray absorption edges. There are currently approximately two dozen synchrotrons producing X-rays for protein crystallography on five continents (there are none in Africa or Antarctica).

In home laboratories (i.e. away from large facilities), low power microfocus X-ray sources developed in the early 2000s gave intensities comparable with a rotating anode, while consuming a few tens of watts rather than kilowatts [11]. More recently, an X-ray source utilizing a liquid target of molten gallium or indium has been developed [12]; this has advantages in always giving a clean target for the electron gun (so the target does not need polishing periodically) and greatly enhanced heat dissipation into the molten metal reservoir (so that the devices can potentially be operated at extremely high power).

## Data collection details. Fine and coarse φ slicing

The vast majority of apparently single crystals are composed of many smaller, slightly misaligned crystal blocks giving rise to an inherent mosaicity, which is often increased substantially during cryocooling. Coupled with imperfect monochromation of the X-rays illuminating them, this means that the data are recorded over a small range of rotation angles even for a stationary crystal. If this range is less than the rotation increment for each image, most of the reflections will occur on single images; this is known as coarse φ slicing or just ‘coarse slicing’ 1. This is most appropriate where the readout time for the detector is significantly longer than the exposure time necessary to record sufficient intensity to measure the spots, for example where the detector is a film or an image plate. For each image, the rotation of the crystal has to be started just before the starting φ so that it has reached full rotation speed by the point the X-ray shutter can be opened. Similarly, the crystal should only begin its deceleration once the shutter is closed. For long exposures of more than a few seconds, the crystal is usually oscillated back and forth, so it is sometimes called the ‘oscillation method’. While the shutter is closed and the image data are being written to disk, the crystal is not rotated. The synchronization of the shutter movement and rotation are critical, particularly when their time scales are comparable.

Conversely, if the diffracting range for the crystal is less than the rotation angle (as is the case for a fast readout detector), the data are termed as ‘fine sliced’; this is the method used mostly today, with detectors such as CCDs and PADs. Provided that the readout time for each image is sufficently small in comparison with the exposure time for each image, the X-ray shutter can be kept open throughout the experiment while continously rotating the crystal, so that the need for accurate synchronization between the mechanical shutter and crystal rotation is eliminated, along with the associated errors; in effect, since the detector is blind when being read out, the readout period is being used as an electronic shutter. This ‘shutterless’ data collection are now the norm at synchrotron beamlines equipped with PADs. Fine slicing has other advantages in that the background measurements can be minimized, and diffraction spots that are close together on the detector can be identified more easily by the integration programs.

## Processing itself

Fortunately for the current generation of structural biologists, the direct electronic recording of the diffraction patterns into image files means they no longer have to work in dark rooms and they do not need to estimate the intensity of each reflection by eye or digitize each photograph in order to prepare the information for subsequent analysis by the data processing programs.

Processing the diffraction images has been dominated by three software packages, all of which can process images from a wide variety of detectors, dating back to the late 1970s to mid-1980s and are not tied to any particular hardware manufacturer. These are Mosflm [13] (distributed as part of the CCP4 suite [14]; scaling and merging is performed by Aimless), HKL2000 [15] (which packages the integration program Denzo and the scaling and merging program Scalepack) and the XDS suite [16,17] (which includes programs for both integration and scaling). A recent initiative has produced a new package, DIALS (see e.g. [18]), which is aimed at data processing at synchrotrons and X-ray free electron lasers (XFELs) in particular; it does not currently have any functionality for scaling or merging. The manufacturers of detectors aimed primarily for the home laboratory have produced their own software as well, but these programs are not usually used for images collected on other manufacturers’ hardware.

Major advances in the field of data processing were made during a series of meetings held in 1986 (the EEC Cooperative Workshop on Position-Sensitive Detector Software) [19]. A standardized parameterization was developed for both the crystal and the instrument, which formalized much of the mathematics behind the method. The FORTRAN code in the existing data processing package MADNES was generalized following these workshops; the underlying methods are still in use in several programs today including the newest development, the DIALS package. d*Trek is a direct descendant of MADNES (recast using modern programming idioms) and the bulk of its code is now freely available [20].

A complicating issue associated with the development of area detectors by a variety of manufacturers is that each has produced at least one (and usually more) unique image file format. Often, the image data are stored in a simple 2-bytes per pixel array, but most image files also have header regions that contain ‘metadata’ which describe the experiment, e.g. rotation range for each image, wavelength of radiation used, crystal to detector distance etc which can be used to help process the data. Unfortunately, the headers do not conform to a single standard and the authors of data integration programs have dealt with this in different ways. For example, XDS skips over the header information and ignores its contents (all information about processing the dataset has to be given in an external control file), Denzo uses a site definition file to hold information about the individual detector which was used for the data collection but makes use of some of the metadata, and Mosflm and DIALS read the header information and use it to set up processing parameters. This means that the metadata needs to be both complete and correct, and this is not always the case. Until recently, all detectors have written each image to an individual file, so a dataset comprising 3600 images will have 3600 files; this has changed with the adoption of the HDF5 container format by Dectris for the Eiger detector, which allows many images to be written on to the same file. The main advantage of using this format is that the process of creating, opening and closing individual files is a time-consuming process that can consequently be largely avoided. The current data processing programs either read the HDF5 files directly or require them to be converted into a more traditional format before processing.

Most detectors write images to file that have been corrected for geometrical distortions and non-uniformity of response, but for the cases where this has not been done, several data processing packages include utilities for this process, as well as for calculating the gain of a detector and overall background level in a dataset, and identifying those parts of the images which should be used for integration (e.g. avoiding masked areas).

Processing X-ray diffraction images collected by the rotation method may be divided conveniently into four different major stages:

1. Spot finding and autoindexing
2. Parameter refinement
3. Integration (the actual measurement of spot intensities)
4. Scaling and merging.

## Spot finding and autoindexing

Indexing is normally carried out using an automated procedure (hence ‘autoindexing’) and gives the crystal orientation, an initial estimate of the unit cell dimensions with an idea of the crystallographic symmetry and an index for each reflection. Before autoindexing can start, a selection of diffraction spots on the images must be located.

The methods for spot finding employed are not well documented beyond the observation that spot finding is extremely slow compared with other stages in process. This lack of speed is largely because the programs are starting with many unknown quantities concerning the diffraction images. At the start of the process, none of the following are known and each needs to be determined:

- background counts on images
- intensity of the spots above background
- their distribution across the images
- the number of useful spots
- the shape of the spots
- the number of pixels that make up each spot.

Since this time-consuming step can be performed independently for each image, the use of multicore processors and multithreaded programming techniques has been effective in reducing the time spent [21]. One important choice at this stage is whether to search for spots on only one or a few images or on many (possibly all) images in a dataset, which may consist of many thousands of images. Using information from multiple images should give extra 3D information, but provided that the crystal does not have one of its principal axes parallel with the incident radiation, and there are plenty of diffraction spots recorded on each image (usually more than approximately 25), indexing is normally possible from a single image.

The default autoindexing in both Mosflm and Denzo operates with spots found on a single image; although each can include spots from multiple images. Mosflm works best with spots from a series of non-contiguous images, which are well separated in φ; by default iMosflm (its dedicated graphical user interface (GUI) [22]) will use spots from two images that are as close as possible to 90° apart in φ [23]. It assigns the midpoint of the rotation range of each image as the value for the reflection in the autoindexing procedure, so if contiguous images are used, different parts of a single reflection may be found on consecutive images, and each part of the same reflection will be assigned a different φ; autoindexing is likely to fail. On the other hand, Denzo can use spots from a sequence of contiguous images (but not from those widely separated in φ). Because it calculates the φ centroid for each reflection that is spread across multiple images, the scattering vectors calculated correspond to a less distorted reciprocal lattice. In practice, both programs are very reliable, and demonstrate that a good sample of spots from one or a few images is sufficient for autoindexing in the vast majority of cases. Other programs combine both approaches, e.g. XDS normally uses a spot search over groups of contiguous images where the groups are well separated in φ; while it can use spots from the entire dataset, most users choose not to, because it is slow and generally unnecessary. DIALS [24] performs a spot search over the entire dataset by default, and is the only commonly used software to do so. Both of these latter two suites calculate the φ centroid for each reflection in a similar fashion to that used by Denzo. DIALS makes further use of the extra information obtained during spot finding in refining the detector and crystal parameters in a later step.

The first step in autoindexing itself is the mapping of the spot positions obtained by projecting the 2D detector co-ordinates on to scattering vectors on the 3D Ewald sphere [25]. A simple transformation for a detector in the symmetric setting has been published [26] but extending this to more generally oriented detectors is rather less straightforward (e.g. [8], chapter 7, especially pp. 82–85). All the scattering vectors calculated in this step correspond to lattice points in the reciprocal lattice.

Indexing routines themselves may be divided into real space and reciprocal space methods. In the former, the search for a unit cell described by the diffraction pattern is carried out after the reciprocal space scattering vectors have been transformed into real space, while the latter performs searches with the scattering vectors directly, converting the results into real space only after successful indexing.

In the early 1990s, Denzo was the first widely available program to have a useful implementation of the Fourier Transform indexing method suggested by Gerard Bricogne at the LURE Phase III workshop [19], and allowed ready indexing from single images as noted above. The robust nature of the algorithm meant that Denzo was used for autoindexing even when another program was used for subsequent refinement of parameters and integration of the dataset. An FFT was calculated on a relatively coarse 3D grid, and a search for three large non-collinear peaks performed to identify a likely primitive unit cell. The grid size for the FFT chosen was small enough for fast calculation, but gives rise to a solution that needs to be refined before determining the primitive triclinic reduced cell and subsequent transformation to higher symmetry Bravais lattices. An independent investigation some years later using a finer grid and the FFT routines in the CCP4 suite was found to be too slow to be of practical use [27].

Until 1998, autoindexing for Mosflm was performed either externally using Denzo or internally using a reciprocal space method (which required multiple images to be reliable) [28]. Subsequently, a 1D FFT method developed as a part of the Data Processing Suite DPS [29] was incorporated into the Mosflm code [26,30]; when coupled with a cell and direct beam position refinement step, this was found to be robust when indexing from a single image, but was considerably more reliable with spots from at least two images widely separated in φ. In this implementation, the scattering vectors calculated from the diffraction spots are projected on to a set of several thousand vectors distributed regularly around a hemisphere of reciprocal space; each projection is Fourier transformed, and a sample of those with the largest non-origin peaks examined to determine if they form a unit cell with reasonable axial lengths and interaxial angles.

DIALS uses spots from the entire dataset by default, and normally uses a real space method employing a 3D FFT indexing routine developed from that used in Labelit [31]; other methods based on 1D FFT and reciprocal space grid search have also been implemented in DIALS but need to be specified when running the program.

The indexing method used in XDS reduces the list of reciprocal lattice points to a few low-resolution difference vectors (i.e. only near neighbours are used in the calculations), and these are accumulated in a 3D histogram which can be searched for large clusters that correspond to reciprocal lattice cell edges. It is particularly successful when used with a good 3D sample of reciprocal space.

Although originally developed for point detectors and small molecule crystallography, DIRAX [32] is also useful for area detector datasets collected on macromolecular crystals; in addition, it has been used for indexing diffraction patterns from incommensurate lattices and for data collected on XFELs [33], where only a single exposure is obtained from every sample. It performs a comprehensive search for periodicity in directions perpendicular to triplets of non-collinear real-space vectors; to avoid false solutions (which may nonetheless score highly) the triplets are formed from random selections of vectors rather than systematically working through the list.

## A note on Bravais lattice determination

Whatever method is used for autoindexing, the initial result is a primitive triclinic cell. All the programs compare the fit of the initial cell with the 44 characteristic lattices listed in *International Tables for Crystallography volume A* [34], via a set of standard matrix transformations. Each of the characteristic lattices (also confusingly called ‘lattice characters’) corresponds to 1 of the 14 Bravais lattices, so each is represented more than once. For example, there are twelve characteristic latttices corresponding to C-centred monoclinic Bravais lattices. Each program uses a different penalty function to describe the goodness-of-fit of the transformed triclinic cell to the appropriate Bravais lattice, e.g.

- Denzo reports a distortion index (as a percentage) based on the mismatch between the calculated and idealized orientation matrices for non-triclinic symmetries and only reports the ‘best’ 14 Bravais lattices [35],
- DIALS uses the Le Page Δ value (in degrees, calculated from angular deviations from the ideal interaxial angles [36]), as implemented in Labelit and cctbx [37], but by default only outputs those characteristic lattices with a value less than 5.0°, flagging those that are particularly good.
- Mosflm and XDS output all 44 characteristic lattices with a penalty for each (or quality index, normalized to a maximum of 999) derived in broadly similar ways from the defining transformations as listed in *International Tables for Crystallography.*

## Parameter refinement

Autoindexing yields an approximate set of parameters that describes the geometry of the unit cell and the diffractometer; usually, the metric symmetry of the transformed unit cell which has a good penalty corresponds to the correct lattice symmetry, e.g. a unit cell obtained from cell reduction that has a = b ≠ c, and all angles 90° is usually tetragonal, but it should be remembered that this is not necessarily the case. Provided that the symmetry is chosen correctly, errors that appear in subsequent processing are due to inaccuracies in mechanical settings (e.g. direct beam position, crystal to detector distance, orientation of the detector and crystal rotation axis) and in the subsequent mapping of the spot co-ordinates to reciprocal space. Where the symmetry has too many constraints (e.g. tetragonal chosen when the true symmetry is orthorhombic), the predicted spot positions will differ markedly from the observed positions that further processing will often fail. This is most easily seen by viewing the original diffraction images with predicted spot positions overlaid, and by identifying any discontinuities in the graphs produced by a GUI. Conversely, using the correct symmetry can lead to more stable parameter refinement.

The agreement indices calculated by the scaling and merging programs (e.g. R_meas_, half-dataset correlation coefficient (CC_1/2_)) are better indicators of the correct symmetry so it is often better to delay making a final choice until after that process has been completed.

Even with the correct symmetry, errors in indexing are important because they result in inaccurate location of the diffraction spots during the integration process. Accurate values are obtained through least-squares refinement, of which there are two approaches in common use.

The most common method, positional refinement, optimizes the fit of observed to calculated spot co-ordinates on the image and is employed by all the integration programs; the second approach analyses the relative intensity of partially recorded reflections across the images on which they appear, to optimize the distance of the reciprocal lattice points from the Ewald sphere. Since this latter method can only be carried out after the intensities of the spots have been measured, it is called ‘post-refinement’. Mosflm uses both as part of its cell refinement stage and also during the integration process. HKL2000 delays post-refinement until after the complete dataset has been integrated; it is carried out in the scaling and merging step by the program Scalepack rather than in Denzo. Similarly, post-refinement in XDS is performed after the integration has been completed, but is used to improve the estimate of the spot intensities rather than to optimize the crystal or detector parameters. DIALS does not use post-refinement, and relies on its robust implementation of least-squares refinement to obtain accurate unit cell parameters.

Positional refinement can be carried out for individual images in isolation (so it is also readily applicable to situations where the dataset consists of images unrelated by a simple rotation about a well-defined axis, e.g. those collected at XFELs); most of the crystal and detector parameters can be refined. Since the values of unit cell dimensions and crystal to detector distance are highly correlated (especially if there are only low-resolution data), care must be taken when they are refined simultaneously. The best way to ensure stability in refinement is to employ a properly weighted eigen value filtering (e.g. by singular value decomposition), which downweights the contribution to refinement of strongly correlated values [38]; running cycles of refinement, alternately fixing and refining correlated parameters is not recommended [39]. The orientation of the crystal about its rotation axis has no effect on this refinement so cannot be refined by this approach.

Post-refinement requires a knowledge of the distribution of the intensities across contiguous images and requires sufficient abutting images to obtain this knowledge. A physical model of how the intensity of the reflections varies with crystal rotation is required – i.e. the ‘rocking curve’; the exact mathematical function used is not critical [40]. Mosflm uses positional refinement to optimize the crystal to detector distance and post-refinement for the unit cell parameters and thus eliminates any correlation.

## Integration

Measurement of the diffracted intensities is termed as ‘integration’ because it is equivalent to calculating the area or volume under a curve. It can be divided into two basic methods: summation integration and profile fitting. Other approaches which are not in common use, include those based around modelling the shape and size of the diffraction maxima from a knowledge of the crystal morphology and orientation, the incident beam shape etc. [41].

It is also possible to integrate ‘two-dimensionally’ where each image is considered separately for the purposes of measurement or ‘three-dimensionally’, where the measurements in an abutting series of images are combined in the measurement stage. Both Mosflm and Denzo utilize 2D integration methods, XDS and d*Trek [42] have implemented 3D profile fitting, and DIALS includes methods for both (a ‘fast 3D profile fitting’ is an option in Denzo but this is not well documented).

Measuring the intensity of diffraction spots is, in essence, a simple process; in the absence of any background counts in the image, the values of each pixel forming a spot on an image can just be added together to give the intensity. The difficulty arises first in locating all the spots accurately (implying correct indexing), identifying which pixels correspond to spots, and taking account of non-zero background on the image (due to detector noise, scatter from other media in the X-ray path etc.). A decision must be made about the level of background under the spot (which can only be measured indirectly) and how to discriminate spot pixels from purely background pixels. The intensity profile of spots is usually a smooth curve that gradually increases to a maximum then decreases; weak spots appear to cover fewer pixels on the detector because the intensity of their shoulders goes below the background level closer to the centroid of the peak than the shoulders of strong spots. From the physics of diffraction, it can be seen that the profiles of neighbouring spots on a detector will be very similar (the cross-section of diffracted rays from source, through the crystal and on to the detector will be similar for reflections with similar indices). Consequently, the profile of strong, well-measured reflections can be scaled and used to improve the estimate of the intensity of nearby weak reflections, giving a better measurement than by just counting the pixels and allowing for background. As a good approximation, any intensity in the shoulders of a strong spot that is obscured by the background will not make a useful contribution to the total measurement. It can readily be shown that profile fitting has little effect on the measured intensities of strong reflections (*c.f.* summation integration), but gives a significant benefit in the measurement of weak reflections on a high background [13].

## Identifying and optimizing the spot region and profile

The region used for integration that distinguishes the spots from the background (the ‘mask’) needs to be optimized in order to maximize the signal-to-noise ratio. Different methods have been employed in the different programs, e.g. Mosflm uses a value called the ‘profile tolerance’, whereby an octagonal mask size for the spot is assumed to have the correct dimensions if the summation integration intensity for a selection of well-measured reflections does not decrease by this fraction if the mask is decreased in size by one row of pixels on opposite edges of the mask.

Other programs use methods based on optimizing the discrimination of background from the diffraction maxima on the detector, e.g. XDS and DIALS find the spot size indirectly by determining where the pixel counts around the spot are only due to the background level; pixels in the region of a spot are sorted on intensity and strong pixels eliminated until the statistical distribution of the intensity of the remaining pixels conforms to that expected for a random sample.

Attempts have been made to formalize the determination of the background region by either calculating the skewness [43–45] or the variance [46] of the intensity distribution, but the methods described have not been widely adopted and are not implemented in any of the commonly used programs. The spot size used in HKL2000 is fixed at the start of processing and is not optimized during integration; if it changes substantially during the course of data collection, integration has to be split into separate runs.

2D integration describes the process where each image is considered individually and the measurement of spot intensities is carried out without direct reference to preceding or subsequent images. When data were collected with slow detectors (e.g. film, image plate, slow CCD or weak X-ray source) the most efficient way to collect data was with coarse φ slicing; most reflections were fully recorded on single images, and 2D integration was considered the best way to process the data, because only a small fraction of spots were partially recorded across two images.

3D integration uses the intensity of reflections spread across several images to build up the measurements, and this is the most appropriate method when the rotation angle for each image is substantially less than the combined mosaicity and beam divergence. Therefore, when data collection is performed with a fine φ slicing method, using a fast readout detector, 3D integration is the method of choice. The optimum degree of fine slicing is partly dependent on the detector used; while approximately a half of the mosaic spread has been found to be optimal for a Pilatus detector [47], for the newer Eiger (which has a substantially faster readout and thus smaller dead-time between exposures), a smaller value of approximately a tenth of the mosaicity has been indicated [48].

The value of the mosaicity itself is defined differently by the programs; Denzo and Mosflm define it as the rocking angle which would generate all the reflections on an image, whereas XDS and DIALS use the S.D. of the reflecting range. In practice, this means that the value reported by XDS or DIALS is approximately two to three times smaller than the value for the same crystal reported by the other programs. Mosflm also allows for the size of the mosaic blocks that make up the crystal; smaller blocks have the effect of increasing the effective mosaicity for the low-resolution reflections while leaving that for high-resolution reflections unchanged [49].

In practice, the major difference between 2D and 3D integration when processing well-collected images from good quality crystals lies in the computing resources that are required for each method, and the results are usually in good agreement. Typically, 2D integration requires fewer calculations than 3D integration, so for a single core computer (as was common until approximately a decade ago) it can be much faster. The development and widespread adoption of multicore CPUs since then, and the parallelization of the code (particularly in XDS) has removed this speed advantage, and has been a major factor in the renewed popularity of 3D integration. DIALS, which has been developed most recently, has been written with the existence of multicore CPUs as a given, and likewise its integration is much faster than single-threaded applications.

On diffraction images, it can be clearly seen that the spot shape varies across the detector and from image to image. In both XDS and DIALS, the shape of the spots on the detector is mapped to an undistorted geometry in 3D space; this means that the effects of the different paths of diffraction maxima passing through the Ewald sphere can be eliminated, as can the effects of obliquity of incidence of each diffraction maximum on the detector.

Other integration programs do not map the shape of the spots in this way. Mosflm and Denzo allow for the varying spot shape and size on the detector by using well-measured spots of medium to strong intensity to calculate an expected profile, but use different approaches to optimize this for different regions of the detector. Mosflm divides the image into either a 3 × 3 (low-resolution images) or 5 × 5 (high resolution) array, and groups together a ‘block’ of adjacent images to provide a good sample of reflections before calculating a profile for each group. For each spot, a weighted profile is calculated from the division in which the spot sits and the three closest divisions. Denzo calculates the profile for each spot based on well-measured spots within the ‘profile fitting radius’ on individual images, which can be optimized to ensure there are sufficient spots for this calculation. XDS allows for different profiles caused by other physical effects by optimizing the size and shape for a central region and eight regions around the periphery of the image.

A more analytical approach to determine the spot shape in integration is based on ray tracing from the X-ray source through the crystal and on to the detector; this is best seen in the program EVAL-15 [41,50] and its predecessor EVAL-14 [51]. Rather than assuming that the spots on the images contain all the information regarding the diffraction experiment, these programs determine the expected spot shape and size on the detector by calculating the 3D reflection boundaries from parameters such as the size, shape and orientation of the crystal, and the cross-sectional profile of the incident beam. EVAL-14 used summation integration, and the code has been developed to employ profile fitting in EVAL-15. Provided that the crystal can be measured accurately (e.g. by face indexing under a microscope) and the physical characteristics of the X-ray source are recorded, this method offers several advantages, e.g.

- profiles can be generated in regions of reciprocal space where there are insufficient, strong, well-measured reflections to develop learnt profiles,
- the effects of spectral dispersion, e.g. Kα1-Kα2 splitting can be taken into account,
- the treatment of convoluted multiple lattices becomes a purely analytical process and diffraction patterns from incommensurate lattices can be measured.

Making the best use of EVAL-15 requires substantial extra care in acquiring the information that is necessary for the ray-tracing algorithm, but this is not always possible, e.g. crystals are often covered with mother liquor and their dimensions cannot be readily measured.

## Scaling and merging

For a variety of physical reasons, the data measured by the integration programs are not on a common scale, and this needs to be taken into account when using the data in subsequent molecular structure analysis. These reasons include changes in incident radiation intensity, illuminated volume of the crystal (e.g. it may precess around the rotation axis, moving in and out of the illuminating beam), anisotropic absorption of X-rays by the crystal, radiation damage to the crystal and non-uniformity of response of the detector itself, so scaling and merging may be thought of as an attempt to model the changes occurring during the diffraction experiment. Because of this role of scaling, the most useful statistics on the self-consistency of the data (merging R values, correlation coefficients etc.) are only obtained after scaling and merging and not directly from integration.

By and large, data integrated by the different programs are scaled and merged by utilities that have been developed alongside; the CCP4 program Aimless [52] is generally used for Mosflm (the program Scala [53] was used previously but is now considered obsolete), Scalepack [15] is used in HKL2000, and XSCALE [54] performs the scaling role for XDS. DIALS has no dedicated scaling program and the data are normally scaled and merged with Aimless.

There are issues in scaling which are relatively easy to correct during integration but which may be very difficult to identify and treat subsequently, largely because of information which has been lost. These are most serious for those reflections with very few symmetry equivalents; if there are only two measurements with widely disparate values, which is most likely to be correct? Two problems in particular involve defective pixels and masked regions of the detector. Defective pixels are either inactive (and always return zero counts) or give unreliably high or low readings (which may vary from image to image). This will only affect those diffraction spots that include those pixels, and their locations on the image should be stored in the individual image files. More seriously, it is difficult in the scaling step to determine if there are shadows on the detector caused by objects like cryocooling devices, the beam stop (or its arm) etc.; these should be masked out at the integration stage, either by using geometric masks or through an intensity threshold where regions of the detector with lower than expected background readings are ignored by the integration program. This latter method is most appropriate for those cases where there is a shadow moving between images, e.g. the shadow of a multicircle goniostat moving across the detector.

There are two aspects to merging reflections; the first merges partials spread over successive images that make up a single measurement, and is only used for 2D integration (since 3D integration gives the intensities of complete reflections directly). The second, more important, aspect is the merging together of multiple measurements of symmetry equivalent reflections, for example for datasets with no anomalous signal the two reflections with indices (h, k, l) and (−h, −k, −l) should have the same intensity according to Friedel’s law; in addition if one considers crystal systems with higher symmetry, e.g. a monoclinic crystal, the intensities of the two reflections related by a two-fold axis with indices (h, −k, l) and (−h, k, −l) should also be the same. The various measures of internal consistency that are reported in the classic ‘Table 1’ in X-ray structural papers are calculated from the mismatch of these equalities from the ideal case.

The measures of data self-consistency are often taken as measures of the overall data quality. Currently, the majority opinion is that the R values and correlation coefficients output by the scaling programs described here are very useful when used to determine whether too much symmetry has been imposed, but less useful in determining the resolution of the data. There should be a clear deterioration in the values for point groups with too much symmetry; analysis of these values calculated from data related by potential rotation axes form the basis of the symmetry tests performed in the program Pointless [53].

Using the data processing statistics (summarized in the Appendix) to estimate the true resolution of the data should be seen as a rule of thumb. Any high resolution limit based on a particular value of I/σ(I), maximum value of R_merge_ or R_meas_ or minimum value of CC_1/2_ is oversimplistic and may exclude useful data which have been measured at higher resolution. The real test is whether the inclusion of extra high resolution data gives more information about the structure than excluding it; for most datasets, the decision is probably best left until the latter stages of structural refinement. Perhaps, the most robust way to determine an appropriate resolution limit for the data is the paired refinement technique, in which the completed structure is refined to convergence with all available data, then further rounds of refinement are performed successively with reduced resolution limits; the resolution at which R_free_ reaches a minimum can be used as a sensible high-resolution limit [55].

The statistics fall into two different groups of measures, neither of which deals with the true quality or information content of the reflections at any particular resolution. For example, for higher resolution reflections, I/σ(I) values are weaker on average than for those at low resolution, so a few strong, well-measured high resolution reflections that contain useful information, may be excluded if a resolution cutoff based on this criterion is applied. Strictly speaking, other measures like R values or correlation coefficients say nothing intrinsically about the quality of the data beyond their self-consistency, but they do provide a useful digest of the processing and data collection, and experience has shown that generally speaking, having a self-consistent dataset is a necessary but not sufficient condition for subsequent work. At the time of writing, much of this is usefully summarized in discussions on the CCP4 bulletin board archives^2^.

## Pipelines and automation

Probably the most important development in data processing in recent years has been associated with that of highly automated beamlines at synchrotrons employing rapid readout detectors such as the Dectris Pilatus (introduced in 2007) and Eiger (available since 2015). The duration of a single data collection has reduced from 5 or 10 min 10 years ago to approximately 5 or 10 s now; whereas it was possible at the turn of the century for a scientist to optimize the processing of each dataset individually, this is no longer the case for all except the most important datasets. Even with data collection times of approximately 10 min, in the region of 100–150 datasets could be obtained in a 24-h shift at a synchrotron, and processing these data while still working on the beamline presented a daunting task for users. Since the early 2000s, this challenge has been addressed and a number of data processing pipelines have been developed which make sensible decisions about processing based on the characteristics of individual datasets. It is possible to integrate datasets in parallel with the data collection, with only a short delay in producing a reflection file and the data processing statistics that are used to infer the quality of the crystal and data collection.

The pipelines use scripting languages such as Python to connect pre-existing data processing programs and to analyse their outputs to optimize processing and avoid false solutions. xia2, probably the most widely used, is an expert system that processes diffraction images with little or no input from the user [56,57]; at synchrotrons it can be triggered to run automatically by the data collection software on the macromolecular beamlines (e.g. at Diamond Light Source). The results from xia2 are presented using a simplified ASCII display which avoids using graphical tools; it is possible at a glance to see if there are discontinuities in the dataset or if problems have arisen. Running xia2 can be quite slow, since it will reprocess data by returning to an earlier stage if it identifies pathologies that require it, for example if the symmetry has been incorrectly assigned too early. AutoProc^3^, which is also run automatically on the beamlines at Diamond has methods and analysis which are broadly similar in scope to those of xia2, but in addition produces a number of graphical tools that are useful in identifying pathologies [58].

Other pipelines have been produced independently at several synchrotrons and have been used to facilitate further automation and remote data collection; examples are FastDP (Diamond Light Source) [59], AutoProcess (Canadian Light Source) [60], xdsme (Soleil) [61] and autoxds (Stanford Synchrotron Radiation Laboratory) [62]; these are all based on scripts that run fast background processing so that users can concentrate on optimizing their data collection experiments, but are only available on beamlines at the corresponding site. Extending the automation pipeline into data collection has resulted in facilities like the MASSIF-1 (Massively Automated Sample Selection Integrated Facility) beamline at ESRF, which is advertized as ‘a beamline that runs itself’; it offers a fully automated facility for characterization and data collection from crystals of biological macromolecules without user intervention [63]. Crystal mounting, centering and data collection can all be performed while the beamline is unattended. Users enter information about the crystals into the sample database so that they can be identified and data collected in accordance with their properties and the particular experiment desired.

From a scientific viewpoint, the principal advantage of automated pipelines is that users have the time to consider the optimum data collection strategy and obtain the best data possible from each crystal, appropriate for the experiment being performed (for example, experimental phasing, molecular replacement, structure refinement, ligand identification etc., each of which places different demands on the data). In practice, rapid beamlines and automated processing have been accompanied by a return to the ‘American method’ of data collection 4 in which crystals are exposed and rotated in the incident X-ray beam with little thought given to the particular information required or the chemical and physical characteristics of the individual crystals.

In the vast majority of data collections performed on macromolecular beamlines at synchrotrons, the automated data processing pipelines yield results as good as most users can get by processing the datasets themselves with the individual programs, and so many users have come to rely on them for routine work. The pipelines tend not to work so well if the crystals suffer from particular pathologies, e.g. they are split or exhibit anisotropic diffraction etc*.* These situations are those where users have to use their expertise to optimize the processing, and illustrate the point that it is well worth the effort invested in learning how to use at least one of the data processing packages.

## User interfaces

The simplest interface, familiar to people working in the 1980s, is the command line that can also be accessed through batch control files, and which traces its origins back to punched cards and paper tape. Automated processing pipelines have promoted the idea of ‘no interface is the best interface’. A good GUI should mean that the user does not need to learn (or consult in a reference manual) processing commands for optimizing data processing. GUIs exist for all the most popular programs, but some are better integrated with their processing packages than others. The HKL2000 interface to Denzo and Scalepack is well integrated and has been developed over many years. A GUI based on a custom graphics library was integrated into Mosflm as a part of the core program in the early 1990s, but a newer, more user-friendly interface called iMosflm has been added since the early 2000s. A number of different graphical interfaces exist for XDS, though none is part of the core package; XDSGUI is best used with a knowledge of XDS commands [64], whereas XDSAPP is based broadly on the appearance and design of iMosflm and can be used without recourse to the core commands [65]. An interface is under development for the DIALS toolkit but has not yet been released for general use [66].

The Mosflm/iMosflm pairing is the only non-commercial combination including a GUI that can be run natively on all commonly available computing platforms (MacOS, Windows, Linux), although DIALS can also be installed on each. XDS is available for Linux and MacOS only, but since the release of Windows 10 can be run via the Windows Subsystem for Linux (WSL) with any of the available GUIs. HKL2000 runs on both Linux and MacOS; since it is released under a licence that ties it to an individual computer, it may not be suitable for running under WSL.

All modern interfaces more-or-less follow a general workflow of reading in diffraction images, finding spots for indexing, indexing itself, refining crystal and detector parameters, integrating the diffraction maxima then either scaling and merging or exporting reflection files for this. Within these tasks, feedback is displayed to help the user to optimize the processing further and enable the user to process data where automated pipelines have either failed completely or have not resulted in a dataset that is useful for subsequent structural analysis. Beyond this, the major differences arise from how this workflow has been implemented. iMosflm is notable in that the interface is particularly uncluttered as a result of the application of modern design principles [67,68].

## Other experiments: Laue diffraction and XFELs

The Laue method, which uses polychromatic radiation and a stationary crystal will give a single image that contains sufficient information which, while not 100% complete, is sufficient to solve many structural problems and is well suited to the study of dynamic processes; the method dates back to the very start of X-ray crystallography. The data at a third-generation synchrotron can be obtained with a single X-ray pulse of as little as approximately 100 ps, so details of molecular processes can be readily studied to that time resolution; trains of pulses can be used to give information at lower time resolution. All these experiments involve an adaption of the pump-probe methodology, which employ some kind of reaction initiation (the pump) followed by the X-ray pulse and recording the image (the probe) [69]. There are currently few options for processing the data, but Precognition appears to be a widely used package [70].

X-ray free-electron lasers can be used for experiments with femtosecond resolution; those in operation include the Stanford Linac Coherent Light Source (SLAC) in the United States and the SPring-8 Angstrom Compact free electron Laser (SACLA) in Japan; others are under construction or currently being commissioned (the European XFEL is due to begin user operation by September 2017, i.e. before this report is published, and the Swiss XFEL is currently undergoing testing). As the X-ray flux for each pulse is extremely high (of the order of approximately 10^12^ monochromatic photons), it is large enough to destroy the sample; fortunately, before destruction, it is possible to generate a diffraction pattern, and a large number of these can be combined to give a complete dataset with extremely high time resolution.

Data integration from diffraction images collected at XFELs has its own issues. While the basic processes are common with the rotation method, there are added complications that arise from the data collection methods used. Independent of the exact method of data collection, the X-ray pulse destroys the sample in a few femtoseconds so that each image is collected from a single crystal in a random orientation. The crystal has almost no time to move during the exposure, so each image is effectively a still (rather than a rotation) image, and consequently the sampling of reciprocal space is only affected by the inherent mosaicity of the crystal and the spectral dispersion of the X-ray pulse; many fewer reflections are recorded per exposure.

While several parameters associated with each exposure can be refined using a positional residual, it is helpful to make use of the greater precision given by post-refinement which needs a measure of the partiality of each reflection. Unlike the rotation method, any measure of the partiality of a reflection has to be estimated along with the mosaicity (which is likely to be different for each crystal) and the known spectral dispersion of each individual X-ray pulse. A method has been developed which estimates the partiality of each reflection based on the initially obtained diffraction geometry and effective mosaicity, followed by iterative refinement to optimize the agreement between these parameters and the calculated intensities for each reflection [71].

Implementations of indexing routines that work with single images (e.g. Mosflm, Dirax) and 2D profile fitting are particularly well suited for working with these data. Because of the huge size of the datasets (many thousands of images), processing is carried out by pipelines that distribute the computing load across many processors, often on multiple machines; examples are Cheetah [72] and CrystFEL [33], originally written to process data collected at SLAC which have since been extended to be used for data collected at SACLA [73]. In addition, the DIALS project has developed new processing tools with the particular properties of XFEL datasets in mind [74].

## Summary

Since the early days of protein structure determination, both the instruments used to collect X-ray diffraction data and the software used to process the data have improved dramatically. The methods of data collection have become so well characterized that they can be programmed into completely automatic processes, and many datasets collected can be processed by automatic expert systems. All these expert systems rely on the quality of the integration and scaling software that has been developed over many years; this software may also be used with well-developed GUIs to process more challenging datasets where the automated pipelines fail, due to unexpected crystal pathologies.

However, the automated data collection process is not perfect, and it should be borne in mind that X-ray data collection is the last experimental step in the single crystal experiment; all subsequent calculations rely on the data being as good as possible. Since it is likely that the process going from identification of the protein target to the point where suitable crystals are available will have taken a considerable amount of time and effort, it seems reasonable to spend a few minutes to identify an appropriate data collection strategy rather than proceed blindly using default settings. Remember that no processing program, no matter how good, can rescue an irredeemably bad dataset.

## Footnotes

1 Although the normal rotation axis of the crystal is usually called a φ axis for an instrument that has only one mechanical axis, and an ω axis for instruments with more, movement about either axis will be referred to as ‘φ rotation’ in this report for brevity.

2 For example, see the discussion in the thread https://www.jiscmail.ac.uk/cgi-bin/webadmin?A2=ind1201&L=ccp4bb&P=R155003&1=ccp4bb&9=A&I=-3&J=on&K=3&d=No+%20Match%3BMatch%3BMatches&z=4 (Accessed 23 September 2017)

3 Registration for use of the pipeline is available at https://www.globalphasing.com/autoproc/ (Accessed 22 September)

4 Rossmann, M.G. and Erickson, J.W. (1983) *J. Appl. Cryst.* **16**, 629–636 ‘We term the new procedure the ‘American method’ for it demands that the shooting be done first and questions asked subsequently. Tradition has it that this was the normal procedure of law enforcement in the American Wild West. Some say traditions die slowly. Certainly it is well suited for dealing with delinquent crystals in the New World of Synchrotrons.’

## Abbreviations

CC: correlation coefficient
CC_1/2_: half-dataset correlation coefficient
DIALS: Diffraction Integration for Advanced Light Sources
ESRF: European Synchrotron Radiation Facility
FFT: Fast Fourier Transform
GUI: graphical user interface
HDF5: Hierarchical Data Format version 5
LCLS: Linac Coherent Light Source
PAD: pixel array detector
SACLA: SPring-8 Angstrom Compact free electron Laser
XFEL: X-ray free electron laser

## Appendix: Quality indices reported in X-ray data processing

This is drawn almost directly from [75] and [76].

Each of these measures is calculated for resolution shells of data and for the dataset overall. Some programs will also calculate the values for reflections binned by intensity.

The value of 〈I/σ(I)〉 (i.e. the average value) for each resolution shell of data will generally decrease as the resolution increases (i.e. becomes smaller numerically, when expressed in Å); historically a value of 2 or 3 was often used as an indicator for an appropriate high-resolution cutoff, partly because refinement programs had trouble working with weak data. Since the introduction of maximum likelihood refinement programs in the mid 1990s, this has no longer been the case.

$$R_{merge}=R_{sym}=R_{linear}=\frac{\sum_{hkl}\sum_{i}\left|I_{i}(hkl)-\langle I(hkl)\rangle \right|}{\sum_{hkl}\sum_{i}I_{i}(hkl)}$$

Where, *I_i_*(*hkl*) = intensity of an individual reflection with indices (hkl)〈*I*(*hkl*)〉 = mean value of the intensity for all reflections with indices (hkl), including those that are equivalent by symmetry.

This is the most widely reported merging statistic; it is less popular now because its value increases when the overall multiplicity of the data increases, even if the precision of their measurement improves.

$$R_{meas}=R_{rim}=\sqrt{\sum_{hkl}\frac{N(hkl)}{\left(N(hkl)-1\right)}}=\frac{\sum_{i}\left|I_{i}(hkl)-\langle I(hkl)\rangle \right|}{\sum_{hkl}\sum_{i}I_{i}(hkl)}$$

Where, *N(hkl)* = the number of reflections with indices (hkl).

All recent scaling and merging programs will report R_meas_, the ‘redundancy independent merging R value’; the multiplier on the left takes account of increased multiplicity in the measurements; it gives a measure of the precision of the individual measurements, and does not increase with higher multiplicity. It is straightforward to show that R_meas_ ∼0.8/〈I_i_/σ_i_〉.

R_merg_ and R_meas_ are measures of the uncertainty for unmerged reflections

$$R_{pim}=\sqrt{\sum_{hkl}\frac{1}{\left(N(hkl)-1\right)}}\times \frac{\sum_{i}\left|I_{i}(hkl)-\langle I(hkl)\rangle \right|}{\sum_{hkl}\sum_{i}I_{i}(hkl)}$$

R_pim_ is almost identical in formulation to R_meas_, differing only in the numerator in the left-hand multiplier. It describes the precision of the averaged merged intensity measurements, and is very useful in determining how well the Bijvoet pairs agree, i.e. those reflections which would be Friedel mates (i.e. have the same intensity) in the absence of an anomalous signal.

It is possible to show that the value of R_pim_ ∼0.8/〈I/σ〉.

$$CC_{1/2}=\frac{\sum_{i=1}^{n}\left(x-\langle x\rangle )(y-\langle y\rangle \right)}{\sqrt{\sum_{i-1}^{n}\left(x-{\langle x\rangle }^{2}\right)}\sqrt{\sum_{i-1}^{n}{\left(y-\langle y\rangle \right)}^{2}}}$$

The CC_1/2_ is a special case of Pearson’s correlation coefficient (CC); rather than calculating the correlation between two independent datasets, a single dataset is divided randomly into two subsets (half the unmerged reflections with indices (hkl) are put into subset x, and half into subset y in the above formulation) and CC is calculated between these. Student’s *t* test can be used to indicate at what resolution there is no longer a significant correlation between the two half-datasets (a value of approximately 0.15 for large datasets).

$$CC^{*}=\sqrt{\frac{2CC_{1/2}}{1+CC_{1/2}}}$$

This is an estimate of the CC of the merged dataset with the true intensities (which are usually immeasurable). Values greater than approximately 0.5 indicate that there is a significant correlation.

It should be noted that CCs are estabished statistical measures, so are widely understood across different fields of study. The R-values are almost entirely confined to crystallography and do not have the same theoretical basis.
