# Correction: X-ray data processing

**DOI:** 10.1042/BSR-20170227C_COR

**Published:** 2020-07-30

**Keywords:** crystallography, data processing, software

The authors of the original article above article “X-ray data processing” (*Biosci Rep* (2017) **37**(5), DOI: 10.1042/BSR20170227) would like to correct the formula given on page 12 for the reliability indices *R_meas_* and *R_rim_*. The author declares that this correction does not change the results or conclusions of the paper. The corrected formula is presented here:.

$$R_{meas}=R_{rim}=\sqrt{\sum_{hkl}\frac{N(hkl)}{\left(N(hkl)-1\right)}}=\frac{\sum_{i}\left|l_{i}(hkl)-\langle l(hkl)\rangle \right|}{\sum_{hkl}\sum_{i}l_{i}(hkl)}$$

